# Surgical Ovarian Suppression and Breast Cancer—What Do We Know About It?

**DOI:** 10.3390/medicina61111905

**Published:** 2025-10-24

**Authors:** Angel Yordanov, Ihsan Hasan, Mariela Vasileva-Slaveva, Eva Tsoneva, Stoyan Kostov, Vesselina Yanachkova

**Affiliations:** 1 Department of Gynecologic Oncology, Medical University-Pleven, 5800 Pleven, Bulgaria; 2 Department of Obstetrics and Gynecology, University Hospital “Sofiamed”, 1504 Sofia, Bulgaria; ihsan_hasanov@abv.bg; 3 Department of Breast Surgery, Specialized Hospital for Active Treatment of Obstetrics and Gynaecology “Dr Shterev”, 1330 Sofia, Bulgaria; sscvasileva@gmail.com; 4 Department of Reproductive Medicine, Specialized Hospital for Active Treatment of Obstetrics and Gynaecology “Dr Shterev”, 1330 Sofia, Bulgaria; dretsoneva@gmail.com; 5 Department of Gynecology, Hospital “Saint Anna”, Medical University—“Prof. Dr. Paraskev Stoyanov”, 9002 Varna, Bulgaria; drstoqn.kostov@gmail.com; 6 Research Institute, Medica University Pleven, 5800 Pleven, Bulgaria; v_ess@abv.bg; 7 Department of Endocrinology, Specialized Hospital for Active Treatment of Obstetrics and Gynaecology “Dr Shterev”, 1330 Sofia, Bulgaria

**Keywords:** breast cancer, ovarian suppression, medical suppression, bilateral salpingo-oophorectomy

## Abstract

Breast cancer (BC) is the most common malignancy in women worldwide, with incidence projected to rise, particularly among younger patients. In premenopausal women with hormone receptor-positive disease, ovarian suppression is an established component of systemic therapy, most often achieved pharmacologically with gonadotropin-releasing hormone agonists (GnRHas). Bilateral salpingo-oophorectomy (BSO) represents a surgical alternative that ensures definitive suppression, eliminates compliance issues, and is more cost-effective in the long term. Despite these advantages, BSO induces irreversible menopause, associated with vasomotor symptoms, cardiovascular morbidity, bone loss, cognitive decline, and reduced quality of life. Evidence suggests that BSO is most appropriate in selected cases, including women unable to tolerate or adhere to medical suppression, those with inadequate estradiol suppression, patients approaching natural menopause, individuals with metastatic hormone receptor-positive disease, and carriers of BRCA1 mutations, especially with triple-negative tumors. Conversely, data on its benefit in BRCA2 carriers remain limited. Overall, BSO provides oncologic outcomes comparable to medical suppression but at the cost of permanent systemic effects. The decision between surgical and medical ovarian suppression should be individualized, balancing oncologic efficacy, comorbidities, genetic background, and patient preference. Further studies are needed to define the optimal duration of medical suppression and clarify the role of BSO in hereditary breast cancer.

## 1. Introduction

Breast cancer (BC) is the most common cancer in women globally [1]. The number of new cases will ultimately continue rising by 2050 [2], with different trends in different age groups [3]. The global incidence of BC will increase the most for women under 50 years [4]. Despite the current advances in BC management, treatment for premenopausal women often requires ovarian suppression. Ovarian suppression using a gonadotropin-releasing hormone agonist (GnRHa) with either tamoxifen or with aromatase inhibitors (AIs) is the standard of care in premenopausal women with hormone receptor-positive BC and a high risk of recurrence [5,6]. This approach is called medical ovarian suppression (MOS). Another approach is the surgical removal of the ovaries—bilateral salpingo-oophorectomy (BSO).

The aim of this review is to investigate in which cases of BC it is appropriate to prefer BSO to MOS.

## 2. Discussion

### 2.1. History of Surgical Ovarian Suppression

The correlation between ovarian function and BC was initially observed by Thomas William Nunn, who documented a case of a perimenopausal women experiencing cancer regression six months post-menstruation cessation [7]. Albert Schinzinger, in 1889, was the first to acknowledge that surgical oophorectomy could be pertinent to BC treatment, having noted that the prognosis for BC patients is more favorable in older women compared to younger ones. He concluded that oophorectomy would accelerate the aging process in younger women, resulting in breast atrophy and diminishing the effects of any pre-existing cancer [8]. The author proposed that this would be effective in both advanced disease and as a preventive measure against local recurrence [8]. These observations were purely theoretical, and Schinzinger never executed the technique. The inaugural bilateral oophorectomy with BC treatment was executed on 15 June 1895 by George Thomas Beatson, subsequently followed by endocrine therapy utilizing thyroid extract [9]. Stanley Boyd was the subsequent individual to execute the surgical procedure, and he was the first practitioner to utilize it as adjuvant therapy [8,10]. The apprehension over surgical consequences and the advent of radiation castration in the early 20th century hindered the widespread adoption of this method [8]. In the 1950s, Charles Huggins and Thomas Dao reintroduced oophorectomy for BC treatment by integrating it with adrenalectomy [11]. In 1992, the Early Breast Cancer Trialists’ Collaborative Group reviewed the existing data and determined that adjuvant oophorectomy, through both radiation therapy and surgical intervention, resulted in enhanced disease-free and overall survival (OS) rates [12]. Subsequent research has shown the advantages of pharmacological (via gonadotropin-releasing hormone agonists) or surgical oophorectomy, particularly in individuals with hormone receptor-expressing malignancies [13,14,15]. By the conclusion of the 20th century, surgical oophorectomy had emerged as a recognized standard method for BC treatment [8].

### 2.2. Current Standard in Ovarian Suppression

Approximately 78 to 85% of newly diagnosed breast cancer cases are hormone receptor-positive [16,17], with 25–30% of these individuals being under 50 years of age at the time of diagnosis [18]. Ovarian suppression, when combined with hormone therapy, has demonstrated efficacy in these patients and enhances survival [19]. It can be executed using GnRHas, supplied subcutaneously on a monthly or tri-monthly basis, through ovarian irradiation, or with BSO [19].

Regarding the age terminology used in this review, we align with major authorities. We use “young women” for those <40 years, consistent with the ESO–ESMO BCY5 consensus on breast cancer in young women [20]. “Perimenopausal” denotes the menopausal transition as defined by STRAW+10 staging (stages −2 to −1), which typically occurs in the mid- to late 40s [21]. “Postmenopausal” is defined clinically as ≥12 months of amenorrhea not due to other causes; globally, most women reach natural menopause between 45 and 55 years (median ≈ 50–51), per the WHO [22]. For clarity when discussing surgery timing, we use “early oophorectomy” to indicate procedures occurring before the usual natural menopause window (i.e., <45–50 years); this is a pragmatic label rather than a formal guideline category. Decisions about ovarian suppression or ablation should be individualized by age and ovarian reserve in line with contemporary ASCO/ESMO recommendations.

The analyses conducted by Francis et al. at 8 and 9 years, respectively, of the Suppression of Ovarian Function Trial (SOFT) and the Tamoxifen and Exemestane Trial (TEXT) focus on the therapy of hormone receptor-positive premenopausal BC patients without BRCA mutations [19]. The findings indicate enhanced overall and recurrence-free survival in patients receiving tamoxifen or AIs who underwent MOS, in contrast to those treated with tamoxifen alone, with superior outcomes observed in the AI cohort (Table 1).

The 8-year overall survival rates for patients who remained premenopausal after chemotherapy in the SOFT study were 85.1%, 89.4%, and 87.2%, respectively. In HER2-negative patients undergoing chemotherapy, the 8-year distant recurrence rate was 7% lower in the SOFT and 5% lower in the TEXT for those receiving an AI in conjunction with MOS, compared to those receiving tamoxifen plus MOS

The 15-year results of the SOFT and TEXT, recently presented at ASCO 2025, confirm that the combination of ovarian function suppression with an aromatase inhibitor provides durable improvements in both disease-free and overall survival compared to tamoxifen alone, with absolute benefits of 4–5% in recurrence-free survival at 15 years [23].

These findings are consistent with earlier evidence from the ECOG 3193 trial [24], which demonstrated similar efficacy between tamoxifen alone and tamoxifen + ovarian suppression in lower-risk patients. Moreover, the ABCSG-5 trial [25] confirmed that goserelin + tamoxifen was comparable to CMF (cyclophosphamide, methotrexate, and fluorouracil) chemotherapy in premenopausal women with hormone-responsive disease.

The last European School of Oncology—European Society For Medical Oncology (ESO-ESMO) guidelines for young breast cancer patients recommend balancing and case-by-case discussion for the method of ovarian suppression in high-risk patients [20].

The incorporation of MOS into treatment elevated the occurrence of grade 3 or higher adverse effects: 24.6% in the tamoxifen-only group, 31.0% in the tamoxifen plus ovarian suppression group, and 32.3% in the exemestane plus ovarian suppression group. Significant uncertainty persists concerning the ideal duration of MOS and its long-term morbidity and mortality implications. It is presumed that the long-term consequences in patients receiving MOS alongside an AI are analogous to those in premenopausal women who have undergone BSO [26].

The updated EBCTCG meta-analysis (2023) encompassing over 15,000 women reinforced the conclusion that ovarian ablation or suppression significantly reduces recurrence and mortality in hormone receptor-positive breast cancer [27].

Research conducted by Oseledchyk et al. indicated that elderly patients are more inclined to receive BSO, which is associated with a minimal risk of complications [26]. Of the 335 women who commenced MOS, 53 subsequently underwent BSO. In a comparison of these 53 women to the 282 who continued solely with MOS, advanced age was the sole factor substantially correlated with the choice to undergo BSO (*p* = 0.035).

Research directly comparing medical and surgical ovarian ablation in women with metastatic BC revealed no difference in progression-free survival (PFS) or OS between the two cohorts [24]. A separate study indicated that BSO, when executed in the adjuvant context, is comparable to the combination CMF when administered with tamoxifen [28,29]. It is important to acknowledge that these studies are outdated, and the treatment criteria for BC have greatly progressed since their publication.

A phase III trial conducted by the Eastern Cooperative Oncology Group (ECOG 3193) [24] and published in 2014 randomized 337 women to receive tamoxifen with or without ovarian function suppression (OFS). OFS was conducted through three approaches: MOS, BSO, or radiation therapy. The research indicated no significant difference in disease-free survival (DFS) (5-year rate: 87.9% vs. 89.7%; log-rank *p* = 0.62) or OS (5-year rate: 95.2% vs. 97.6%; log-rank *p* = 0.67) between the two cohorts. It is important to note that the study population comprised low-risk patients—premenopausal women with node-negative, estrogen receptor (ER)-positive and/or progesterone receptor (PgR)-positive primary invasive BCs with tumors measuring ≤3 cm in diameter. The group receiving OFS exhibited an increase in menopausal symptoms, a decline in sexual activity, and a deterioration in health-related quality of life at the 3-year follow-up. In the OFS group, 22.4% of patients experienced grade 3 or higher adverse events, whereas the tamoxifen-only group reported 12.3%. Significantly, 42% of patients in the OFS group underwent BSO. The authors observed no variations in disease-free survival or OS related to the type of ovarian function suppression, nor in quality of life or complication rates across the various subgroups. They reported a statistically significant negative impact on sexual function in the BSO group, though this finding was derived from a small sample size [24].

Ferrandina et al. conducted a study assessing the cost-effectiveness of laparoscopic BSO compared to GnRHa administration in patients aged 40–49 years with hormone-sensitive BC. The findings indicated that the surgical procedure is more cost-effective in the adjuvant setting [30]. Another study reported similar findings, indicating that after two years of goserelin use, oophorectomy becomes more cost-effective [31].

Suh et al. performed a direct comparison of MOS and BSO in a cohort of 66 premenopausal patients diagnosed with hormone receptor-positive, recurrent or metastatic BC who were receiving treatment with AIs [32]. Despite the lack of statistical significance, the surgical group exhibited a numerically higher clinical benefit rate (CBR) of 88% compared to 69% (*p* = 0.092) and a longer PFS of 17.2 months versus 13.3 months (*p* = 0.245) [26]. It is important to acknowledge that the limited sample size constrains the robustness of these findings.

At present, there are limited studies that directly compare medical and surgical options for OFS. From the available data, the following conclusions can be derived: ovarian suppression, when combined with SERMs or AIs, enhances survival; however, there is insufficient evidence to indicate that oophorectomy provides a distinct benefit [33]. This necessitates a careful and tailored approach to its application. The emergence of molecular classification in BC and the integration of novel treatment approaches, including neoadjuvant chemotherapy and CDK4/6 inhibitors, complicate the extrapolation of findings from previous studies to contemporary clinical practice.

### 2.3. Effectiveness of Ovarian Suppression with Medications

GnRHs do not consistently achieve full estradiol suppression. This may result from multiple biological, pharmacotherapeutic, and laboratory aspects [34]. Prolonged non-pulsatile injection of GnRH is known to cause downregulation of GnRH receptors in the pituitary gland, resulting in decreased production of follicle-stimulating hormone (FSH), luteinizing hormone (LH), estradiol, and progesterone from the ovary. Nonetheless, various treatment protocols exert distinct influences on FSH and LH levels. When GnRH is delivered in isolation, LH is entirely repressed without recovery, whereas FSH levels, initially depressed, gradually increase and may often return to baseline levels. This recovery is elucidated by the mechanism of inhibin. Consequently, while ovarian stimulation diminishes and hydration is crucial, the individual responsiveness of the ovaries to GnRH agonists is paramount, mostly influenced by age. Women under 40 possess a superior ovarian reserve and a more intact hypothalamic–pituitary–gonadal axis. In some women, the pituitary gland may not be adequately inhibited to curtail ovarian hormone production; although estradiol synthesis is diminished, this inhibition is not enduring. The ovaries can demonstrate a phenomenon of “escape” from the suppressive action, particularly in younger women with superior reserve. The findings of a prospective study conducted by Tesch et al. indicate that, among young women under 40 years of age receiving GnRH treatment, around 5% had an elevated E2 suppression threshold of 10 pg/l [6,35]. The research conducted by Luo et al. elucidates the phenomena of ovarian “escape” as a diagnostic and therapeutic problem, emphasizing the absence of standardized protocols for the treatment and monitoring of these individuals [36]. A factor that diminishes the responsiveness to GnRH suppression is the patient’s body mass index. Individuals with an elevated body mass index exhibit markedly steady and elevated estradiol levels despite suppression, attributable to the peripheral aromatization of adrenal androgens within adipose tissue [37].

The use of various regimens, suboptimal dosage, and variations in the pharmacokinetic characteristics of the medications are also significant. Long-acting medications are unable to sustain consistent levels of ovarian suppression, particularly in individuals with overweight, obesity, or comorbidities that affect the pharmacokinetics of the drug.

A summary of the main studies evaluating the effectiveness of medical ovarian suppression is presented in Table 2.

#### 2.3.1. Anti-Müllerian Hormone (AMH) as a Biomarker for Ovarian Reserve and Guidance in Ovarian Suppression Decisions

Anti-Müllerian hormone (AMH) is a dimeric glycoprotein produced by granulosa cells in preantral and small antral follicles. It signifies the existing or surviving follicular pool and functions as a sensitive measure of ovarian reserve [38]. AMH may serve as a biomarker for the potential preservation of fertility in premenopausal women with hormone-positive breast cancer undergoing ovarian suppression, as well as a prediction of chemotherapy-induced ovarian failure. Research findings indicate that pre-chemotherapy or suppressive treatment AMH levels are significantly associated with the probability of ovarian recovery following treatment termination. Patients exhibiting elevated baseline AMH levels are more inclined to regain ovarian function and menstruation post-chemotherapy, while undetectable or significantly low hormone levels correlate with a substantial likelihood of irreversible ovarian failure [39,40]. Li Y. et al. confirmed a significant reduction in AMH levels following cytotoxic therapy in their meta-analysis, thereby endorsing its potential as an early indicator of gonadotoxicity [41]. Moreover, Van Zwol-Janssens et al. shown that the integration of pretreatment AMH and patient age enhanced the prognosis of ovarian function recovery in comparison to chronological age alone [42]

In clinical practice, AMH may offer further insights for determining a therapy regimen for patients necessitating extended ovarian function suppression. Post-chemotherapy, undetectable AMH levels, together with diminished estradiol and heightened FSH, may indicate irreversible ovarian failure, thus permitting the safe administration of aromatase inhibitors without concurrent ovarian suppression [43]. In contrast, detectable but low AMH levels post-therapy suggest relatively maintained follicular activity and a potential risk of adequate estradiol production, necessitating ongoing suppression with GnRH agonists or the consideration of bilateral salpingo-oophorectomy [43]. Recent studies suggest that anti-Müllerian hormone (AMH) may serve as a biomarker of ovarian reserve and could help identify women at risk of incomplete suppression [36,44]. Nonetheless, AMH is not regarded as an independent diagnostic or monitoring instrument for assessing the effectiveness of ovarian suppression. The serum concentration decreases swiftly upon the commencement of GnRH agonist therapy and may not accurately indicate the extent of estradiol suppression throughout treatment. The measurement of estradiol continues to be the benchmark for evaluating the adequacy of suppression [23,45].

Notwithstanding these constraints, AMH may function as a predictive tool in customizing the treatment strategy for women receiving such medication, facilitating the identification of those at risk for inadequate ovarian suppression or recovery, and enhancing pre-treatment fertility counseling.

#### 2.3.2. Global and Ethnic Perspectives on Ovarian Suppression and Biomarkers

International guidelines (ASCO/NCCN, ESO–ESMO BCY5) consistently advocate for ovarian suppression, typically utilizing a GnRH agonist, tamoxifen, or an aromatase inhibitor, for premenopausal women diagnosed with hormone receptor-positive breast cancer at moderate to high risk of recurrence [20]. Surgical ovarian ablation (SOA) is advised when pharmacological suppression is intolerable, when patient adherence is inconsistent, or when prolonged access to GnRHa is restricted, a situation frequently encountered in low- and middle-income nations. Ethnic and regional disparities affect both baseline AMH levels and therapeutic approaches. Black and Hispanic women exhibit lower mean AMH concentrations, with greater variability compared to Asian populations. This indicates significant demographic disparities in ovarian reserve and chemotherapy-induced gonadotoxicity [46]. The disparity in access to GnRH agonists, aromatase inhibitors, and laboratory hormonal tests contributes to the global variations in ovarian suppression utilization. Consequently, the BHGI and NCCN guidelines endorse BSO as a pragmatic, cost-efficient option when prolonged medical suppression is impractical [47].

### 2.4. Quality of Life in Menopausal Patients

Inducing menopause, irrespective of the method employed, results in distinct symptoms and a decline in quality of life. Patients with BC who are induced into menopause reportedly experience more severe symptoms compared to those who undergo natural menopause [48,49,50].

Surgical menopause causes sudden and irreversible changes in how the ovaries function. This rapid shift may be connected to sudden, and in some cases severe, side effects of ovarian endocrine malfunction, such as neurovegetative symptoms, cognitive deficits, cardiovascular diseases, and a very fast reduction in bone density.

Menopause caused by medication is different from menopause caused by surgery, and in some cases, it may even be reversible. There are several clinical signs of drug-induced menopause, and the severity of these signs depends primarily on the patient’s age and the exact medicine they are taking. Chemotherapy and radiation therapy often cause delayed, subtle changes in how the ovaries work. These changes happen more often than expected. GnRH analogues work faster to stop ovarian function, although the changes they make are often reversible if the ovarian tissue is still intact.

Yet, regardless of whether the main intervention is surgery or medication, menopause caused by either must be treated with a highly individualized approach, depending on the disease and the patient’s condition. The main goal of treatment is to lower the risks to heart health, bone health, and brain function [51].

Demir O. et al. performed a study on women experiencing natural and surgically induced menopause utilizing the Menopause Rating Scale. The findings indicated that women experiencing surgical menopause exhibited a significantly (*p* < 0.05) greater prevalence of physical and psychological symptoms in comparison to those undergoing natural menopause [52].

Surgical menopause increases cardiovascular risk twofold in women under 50, associated with abrupt alterations in cardiometabolic parameters [53].

Research conducted by Prince et al. indicated that women experiencing surgical menopause exhibited elevated mean Framingham Risk Score levels in comparison to those undergoing natural menopause [54]. Chemotherapy and radiation therapy can change cardiometabolic parameters, especially when ovarian function is lost for a long time.

Estradiol levels drop a lot, which quickly lowers bone density. This is especially true for women who are going through surgical menopause or drug-induced irreversible menopause. These women need early intervention to make sure their bone health is protected [51].

Early oophorectomy is positively associated with the development of dementia, a heightened prevalence of parkinsonism, and increased rates of sadness and anxiety. Endocrine therapy, on the other hand, is not associated with an increased risk of developing dementia in breast cancer survivors [55].

Both forms of induced menopause result in diminished libido and sexual dysfunction due to hormonal alterations and consequent vaginal atrophy. In medically induced menopause, the symptoms are milder and less severe [54].

Women under 40 years typically have higher ovarian reserve and may experience incomplete suppression with GnRHas; for example, in a cohort of breast cancer patients, age ≥ 40 was significantly associated with persistent chemotherapy-induced ovarian failure [56]. Between 40 and 49 years, the physiological transition toward menopause may influence the risk–benefit balance of suppression, as suggested by differential outcomes in women under 45 in long-term analyses [27]. Current ASCO/ESMO guidance and recent surveys underscore the importance of individualized OFS strategies tailored by age, ovarian reserve markers, and patient risk factors [6,57].

Robust data specific to BC patients are lacking; however, it is well-established that adnexectomy, especially prior to age 45, elevates the risk of cardiovascular mortality [33]. BC patients who undergo oophorectomy exhibit an increased risk of fractures, as they typically experience greater bone mass loss compared to those who do not undergo the procedure [33]. Surgical castration adversely affects overall quality of life by inducing vasomotor, psychological, and sexual symptoms, potentially hindering treatment adherence [33]. Patients with severe symptoms resulting from medical suppression may receive a temporary “drug holiday” or therapeutic adjustment for symptom relief; however, this option is unavailable for those who have experienced surgical menopause.

Studies directly comparing the side effects of various methods of OFS in BC are insufficient. Boccardo et al. conducted a study comparing side effects in patients with metastatic BC treated with goserelin and those who underwent oophorectomy. The research indicated an increased occurrence of adverse effects in the group receiving medical treatment [58].

Oophorectomy, regardless of age, significantly elevates the risk of all-cause mortality. Consequently, the choice to undertake the procedure should be meticulously evaluated in relation to the risks of recurrence and cancer-specific mortality [33].

The key studies comparing quality of life outcomes between surgical and medical menopause are summarized in Table 3.

### 2.5. Ovarian Suppression in BRCA1/2 Carriers

Prophylactic BSO in carriers of BRCA1 and BRCA2 mutations is recognized to decrease the risk of BC development [59]. Despite that, two recent studies unexpectedly reported no decrease in BC risk after oophorectomy in BRCA1 carriers [60,61]. This prompts a further evaluation of the role of BSO in individuals with BRCA1 and BRCA2 mutations who have already been diagnosed with BC.

The initial investigation was carried out by Metcalfe et al., who examined a cohort of 676 carriers of BRCA1 and BRCA2 mutations diagnosed with stage I or II BC [62]. In BRCA1 mutation carriers, oophorectomy, whether conducted prior to or following diagnosis, was associated with a 60% reduction in BC mortality. The hazard ratio (HR) was 0.27 (95% CI, 0.11–0.66; *p* = 0.004) when the intervention was conducted within two years of diagnosis. The findings in BRCA2 carriers were not statistically significant (HR, 0.57 [95% CI, 0.23–1.43]; *p* = 0.23) [63]. Mortality was significantly reduced in patients with triple-negative BC (HR, 0.07 [95% CI, 0.01–0.51]; *p* = 0.009). Valentini et al. conducted a study involving 397 young women diagnosed with BC and BRCA1 mutations, revealing that oophorectomy correlated with an 80% decrease in mortality [63]. Huzarski et al. found a 70% decrease in BC mortality following oophorectomy, accounting for additional treatments and prognostic factors in BRCA1 mutation carriers. The limited occurrence of BRCA2 in their cohort precluded any conclusions about the significance of BSO in BRCA2-positive patients [64].

In 2010, findings from a prospective, multicenter cohort study involving 2482 women with BRCA1 or BRCA2 mutations indicated that oophorectomy decreased BC-specific mortality by 73% in BRCA1 carriers (HR, 0.27 [95% CI, 0.12–0.58]), while no significant effect was observed in BRCA2 carriers. The procedure demonstrated a significant reduction in overall mortality among BRCA1/2-positive patients with a history of BC (HR, 0.30 [95% CI, 0.17–0.52]) [65].

The available data allows for the following conclusion: Carriers of BRCA1 mutations diagnosed with BC may experience several advantages from early oophorectomy, such as a lower incidence of second primary ovarian cancers and a reduction in BC-related mortality [45]. Certain authors propose that the procedure be conducted within one year of diagnosis to enhance outcomes [66].

Comparative findings of the main studies evaluating BSO outcomes in BRCA1/2 mutation carriers with breast cancer are summarized in Table 4.

Benefit–risk assessment is crucial when evaluating BSO for BC patients. BSO elevates the risk of all-cause mortality, modifies quality of life, and induces irreversible menopause, leading to complications such as hot flashes, vulvovaginal atrophy, and bone loss. Ovarian suppression should be implemented promptly when indicated, and knowledge of BRCA status is essential, as BSO markedly enhances prognosis in BRCA1-positive triple-negative patients.

## 3. Open Questions and Future Directions

Despite significant progress, several key questions remain unresolved. The optimal duration of medical ovarian suppression and its equivalence to surgical castration require clarification through long-term prospective studies. The role of AMH and other biomarkers in monitoring ovarian function suppression is evolving and should be validated in larger, ethnically diverse populations. Additionally, the impact of early versus delayed BSO on quality of life, cognition, and metabolic outcomes warrants further evaluation. Future clinical trials integrating genomic profiling, BRCA status, and endocrine dynamics may allow more individualized ovarian suppression strategies.

**Figure 1:**
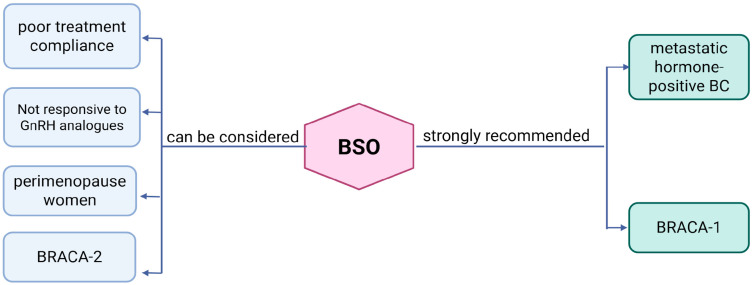
Indications for BSO in BC patients.

The following conclusions can be drawn from the available data (Figure 1):

BSO is suitable for patients with hormone receptor-positive BC who cannot comply with GnRHa protocols.

BSO is a viable option for hormone-positive patients when medical suppression has proven ineffective (when estrogen levels are not low enough), including patients with overweight, obesity, or comorbidities that affect the pharmacokinetics of GnRHas.

- BSO is suitable for patients with hormone receptor-positive BC nearing natural menopause.
- BSO is appropriate for patients with metastatic hormone receptor-positive BC.

BSO is indicated for BC patients with BRCA1 mutations and may also be relevant for those with BRCA2 mutations.

## 4. Conclusions

Ovarian suppression is an essential component of adjuvant therapy in premenopausal women with hormone receptor-positive breast cancer. While medical ovarian suppression with GnRHa remains the current standard, bilateral salpingo-oophorectomy represents a safe, effective, and cost-efficient alternative in selected cases. BSO may be particularly appropriate for patients who are unable to adhere to or respond adequately to medical suppression, those approaching natural menopause, and women with metastatic disease. In BRCA1 mutation carriers, especially with triple-negative tumors, BSO is associated with a significant survival benefit and should be strongly considered. However, the irreversible induction of menopause and its systemic consequences necessitate careful evaluation of risks and long-term morbidity. Future research should clarify the optimal duration of medical suppression, refine patient selection, and better define the role of BSO in BRCA2 mutation carriers. Ultimately, the choice between medical and surgical ovarian suppression must be individualized, balancing oncologic benefit, quality of life, and patient preference.

## Figures and Tables

**Table 1:** Results for overall and recurrence-free survival (analysis of the tamoxifen or AI groups who underwent MOS is biased and is also for 8 years).

Study	Overall Survival			Disease-Free Survival Rate		
	Tamoxifen	Tamoxifen Plus Ovarian Suppression	AIs Plus Ovarian Suppression	Tamoxifen	Tamoxifen Plus Ovarian Suppression	AIs Plus Ovarian Suppression
SOFT (8 years)	91.5%	93.3%	92.1%	78.9%	83.2%	85.9%
TEXT + SOFT (8 years)		93.3%	93.4%		82.8%	86.8%

Aromatase inhibitors—AIs.

**Table 2:** Summary of studies evaluating the effectiveness of medical ovarian suppression (MOS) in premenopausal breast cancer.

Study (Year)	Population	Intervention	Main Findings
Tesch et al., 2024 [35]	Premenopausal women <40 years on GnRHa	Estradiol suppression thresholds	~5% had inadequate E2 suppression (escape phenomenon)
Luo et al., 2025 [36]	Premenopausal BC on GnRH	Review/analysis	Described “ovarian escape” and need for standardized monitoring
McCann et al., 2024 [37]	ER+ BC, on GnRHa	Biomarker assessment	Highlighted variability in E2 suppression by BMI and drug kinetics
Dowsett et al., 2016 [34]	Early BC, premenopausal	Clinical commentary	Incomplete estrogen suppression may reduce efficacy

**Table 3:** Comparative data on quality of life, cardiovascular, and bone effects after induced menopause in breast cancer patients.

Study (Year)	Type of Menopause	Key Outcomes	Main Conclusion
Demir et al., 2020 [52]	Surgical vs. natural	Higher physical/psychological symptoms in surgical group	Surgical menopause significantly worsens QoL
Secoșan et al., 2019 [53]	Surgical	Review	Increased risk of CVD and bone loss after oophorectomy
Price et al., 2021 [54]	Early/surgical vs. natural	Framingham Risk Score	Early/surgical menopause doubles CVD risk
Boccardo et al., 1994 [58]	GnRHas vs. oophorectomy	Side effects	Slightly more side effects with medical suppression
Tan et al., 2025 [55]	Post-BC survivors	Dementia risk	Endocrine therapy not linked to dementia risk

**Table 4:** Summary of key studies evaluating oophorectomy outcomes in BRCA1/2 mutation carriers.

Study (Year)	Population	Key Findings	Effect on Breast Cancer Mortality
Metcalfe et al., 2015 [62]	676 BRCA1/2 carriers	Oophorectomy within 2 yrs ↓ mortality (HR 0.27) in BRCA1	Significant reduction (60%) in BRCA1; NS in BRCA2
Valentini et al., 2013 [63]	397 BRCA1+	Oophorectomy ↓ mortality by 80%	Strong benefit for BRCA1
Huzarski et al., 2013 [64]	BRCA1+	70% decrease in BC mortality	Confirmed survival benefit
Domchek et al., 2010 [65]	2482 BRCA1/2	↓ BC-specific mortality 73% (BRCA1 only)	No effect in BRCA2
Kotsopoulos et al., 2017 [60]	Healthy BRCA1/2	No decrease in BC risk after oophorectomy	Reevaluation needed

The arrow (↓) means decrease.

## Data Availability

The authors declare that all related data are available from the corresponding author upon reasonable request.
